# Functional characterization of COMT genes in Chinese white pear (*Pyrus bretschneideri*) and their role in lignin synthesis

**DOI:** 10.3389/fpls.2025.1614220

**Published:** 2025-06-09

**Authors:** Xiaofeng Feng, Xianang Wu, Jiayi Hong, Zihui Guo, Yasmin Khan, Dongyi Wei, Honghong Fan, Yongping Cai

**Affiliations:** ^1^ School of Life Sciences, Anhui Agricultural University, Hefei, China; ^2^ Characteristic Plants, Anhui Agricultural University, Hefei, China

**Keywords:** Chinese white pear (*Pyrus bretschneideri* Rehd), COMT, Dangshan Su, lignin, stone cell

## Abstract

**Background:**

Caffeic acid O-methyltransferase (*COMT*) is an S-adenosyl-L-methionine (SAM)-dependent O-methyltransferase that catalyzes the methylation of caffeic acid to form ferulic acid, a critical step in lignin biosynthesis. Lignin is essential for the development of stone cells in pear fruit, imparting their characteristic texture. Although *COMT* has been extensively studied in model organisms, its function in pears remains less explored.

**Results:**

In this study, we identified 29 *COMT* genes in the pear variety ‘Dangshan Su’, classified into five subfamilies. These genes exhibit five conserved motifs, and promoter analysis indicates potential hormonal regulation. Transcriptome data showed that *PbrCOMT1* is the predominant COMT gene in ‘Dangshan Su’ fruit and is likely crucial for lignin synthesis. *In situ* hybridization revealed that the expression of *PbrCOMT1* coincides with lignin, highlighting its role in stone cell development. Functional studies, including transient transformation of pear and strawberry fruit, as well as stable transformation of *Arabidopsis* thaliana, demonstrated that *PbrCOMT1* overexpression enhances lignin content, while gene silencing diminishes it. Overexpression in *Arabidopsis* and fruit models resulted in growth inhibition, associating *PbrCOMT1* with lignin-related developmental processes.

**Conclusions:**

Our findings indicate that *PbrCOMT1* is a key gene involved in lignin synthesis and stone cell development in pear fruit. This provides a molecular basis for enhancing pear fruit quality through targeted genetic approaches.

## 1 Introduction

Pear (*Pyrus L.*), a significant woody perennial fruit tree of the Rosaceae family, is widely distributed globally (Wu et al., 2013). Originating in China, pear exhibits considerable genetic diversity and dominates global production, representing over 70% of the world’s cultivated area and production (Wu et al., 2018). The quality of pear fruit is adversely affected by stone cells, which form clusters in the pulp (Cai et al., 2010; Cheng et al., 2018). Larger and more abundant clusters of these cells detract from the quality of the pear pulp, underscoring the importance of their size and concentration in determining fruit quality (Xue et al., 2020; Zhang et al., 2021; Cao et al., 2025).

Stone cells develop through the accumulation of lignin in the cell walls of parenchyma cells, culminating in the deposition of a secondary cell wall (SCW) (Choi et al., 2007; Yan et al., 2014). Thus, exploring lignin synthesis in pear fruit is vital for understanding stone cell formation and developing strategies to regulate their number, aiming to enhance pear quality.

The development of pear fruit, in relation to stone cell development, can be segmented into four periods: pre: up to 15 days after flowering (DAF), when thin-walled cells begin to split; middle: 15 DAF to 23 DAF, during which the secondary wall thickens and stone cells start to form and aggregate into masses (Su et al., 2019); late: 23 DAF to 67 DAF, when the stone cell mass peaks at 67 DAF; end: post 67 DAF until fruit ripening, where thin-walled cells around the stone cell mass expand into elongated and oval shapes, and the content of the stone cell mass decreases (Choi and Lee, 2013; Tusong et al., 2022).

Stone cell distribution in pear fruit is uneven and changes markedly during the growth period (Li et al., 2020). In ‘Dangshan Su’ pears, stone cell content increases from 15 to 55 DAF, with a significant rise between 35 and 55 DAF, resulting in a high stone cell density in the pulp. From 55 DAF to maturity, as the fruit expands, stone cell density declines and becomes concentrated near the core. Observations indicate that stone cells are denser near the peel and core compared to the pulp (Wang et al., 2023; Gong et al., 2023).

Lignin synthesis proceeds through three stages:

1. Shikimate pathway: Photosynthetic products generate phenylalanine, tyrosine, and tryptophan.
2. Phenylpropanoid pathway: Lignin monomers are produced through hydroxylation, methylation, and reduction of phenylpropanoid monomers.
3. Polymerization: Various lignin monomers polymerize to form lignin (Zhao, 2016; Cheng et al., 2019).

There are three primary types of lignin monomers: Syringyl lignin (S-lignin), derived from syringyl propane units; Guaiacyl lignin (G-lignin), originating from guaiacyl propane units; and Para-hydroxyphenyl lignin (H-lignin), produced from para-hydroxyphenyl propane units (Boerjan et al., 2003; Wang et al., 2013).

Differential methylation of aromatic rings characterizes these monomers. *CCoAOMT* and *COMT* are key enzymes in lignin biosynthesis (Fornale et al., 2017).

*COMT* is particularly critical for the O-methylation at the C5 position of the phenolic ring, facilitating the conversion of caffeic acid to ferulic acid and 5-hydroxyconiferaldehyde or 5-hydroxyconiferyl alcohol to sinapaldehyde or sinapyl alcohol. This process, essential for forming G and S units of lignin, has been observed in *Arabidopsis thaliana* (Guo et al., 2001; Lam et al., 2007). A lack of *COMT* impairs plant growth and reduces flavonoid lignin units, resulting in a “brown midrib” leaf phenotype in maize (Fornale et al., 2017). Conversely, in *COMT* knockout/deletion mutations, benzodioxane substructures are detected in *Arabidopsis* thaliana, oilseed rape, and poplar, attributable to the presence of 5-hydroxyconiferyl alcohol (5-OH-CA), a rare substance in normal plants (Lu et al., 2010; Oraby and Ramadan, 2015; Moinuddin et al., 2010). It has been demonstrated that *COMT* is highly expressed in pear fruit, and changes in its expression correlate with lignin levels, suggesting that *COMT* is likely to play a critical role in the development of stone cells (Cao et al., 2019; Mamat et al., 2019).

In this study, we screened and identified the *COMT* gene in Chinese White Pear (*Pyrus bretschneideri*) and analyzed the gene and protein structures, including the cis-acting elements on the *COMT* promoter. The role of *PbrCOMT1* in lignin synthesis and stone cell development was investigated through *in situ* hybridization in pear fruit, transient transformation in both pear and strawberry fruit, and stable transformation in *Arabidopsis*.

## 2 Materials and methods

### 2.1 Identification of COMT genes in *Pyrus bretschneideri*

Genomic data, including CDSs, protein sequences, and gene annotation files (in GFF/GFF3 format), were accessed from five Rosaceae species—*Pyrus bretschneideri*(GCF_000315295.1) (Wu et al., 2013), *Fragaria vesca*, *Malus × domestica*, *Prunus mume*, and *Prunus avium*—via the Rosaceae Genome Database (GDR) (https://www.rosaceae.org/). A local database was created using DNATOOLS software, incorporating the amino acid sequences of the *Pyrus bretschneideri COMT* gene (Cao et al., 2019).

Following the method described by Molinari, the Methyltransf_2 family structural domain (PFam: PF00891) was used as the query sequence. Following the method described by Molinari et al., the Methyltransf_2 family domain (Pfam accession: PF00891) was used as a query. Candidate *COMT* genes were identified from the Pyrus bretschneideri genome using DNATOOLS software, applying an E-value threshold of 0.001(Cao et al., 2024b). Candidate sequences were further analyzed for conserved domains using SMART (http://smart.embl-heidelberg.de/) and Pfam (http://pfam.xfam.org/) to confirm the presence of *COMT* family domains. Additionally, the molecular weight and isoelectric points of the identified *COMT* proteins were calculated using the ExPASy online tool (http://web.expasy.org/protparam/).

### 2.2 Phylogenetic analysis

All COMT protein sequences were aligned using ClustalW in MEGA 11.0. A phylogenetic tree was constructed using the Neighbor-Joining (NJ) method with 1,000 bootstrap replicates to assess branch support.

### 2.3 *COMT* gene structure and conserved motif prediction

Gene structures were analyzed using the Gene Structure Display Server (http://gsds.cbi.pku.edu.cn) to compare the arrangements. Conserved motifs in *PbCOMT* protein sequences were identified using the MEME Suite (http://meme-suite.org/tools/meme). The search parameters were set to identify a maximum of 5 motifs, with motif widths ranging from 6 to 200 amino acids.

### 2.4 Cis-acting element analysis in *Pyrus bretschneideri COMT* gene promoters

Promoter sequences spanning 2000 bp upstream of the start codon (ATG) for each *COMT* gene were retrieved from the *Pyrus bretschneideri* Genome Database. Analysis of the cis-acting elements within these promoter regions was conducted using the PlantCARE tool (http://bioinformatics.psb.ugent.be/webtools/plantcare/html/) (Cao et al., 2024a).

### 2.5 Comparison of pear transcriptome data with reference genomes

Transcriptome data for pear fruit at various developmental stages in this study are available in the SRA database under accession numbers SRR5965142, SRR5965144, and SRR5965146. Transcriptome data for different tissue types can be accessed through the Pear Multiomics Database (https://pearomics.njau.edu.cn/) (Hu et al., 2023). The reference genome is available at https://www.ncbi.nlm.nih.gov/sra. Gene annotation of the transcriptome was carried out using pear genome data (https://www.ncbi.nlm.nih.gov/datasets/genome/GCF_000315295.1/). The expression levels of individual genes were quantified by calculating fragments per kilobase of transcript per kilobase of exon model per million mapped reads (FPKM), which were used to assess expression patterns.

The FPKM values of *COMT* family genes across different tissue types and developmental stages of pear fruit were normalized using the normalize function for column scaling. A heatmap was then generated, and hierarchical clustering was applied to the rows of the heatmap to analyze gene expression patterns.

### 2.6 Plant materials

This investigation used ‘Dangshan Su’ pears grown in the erstwhile Yichang Agricultural Park, Dangshan County, Anhui Province, China. For the experimental procedures, pears that were 39 DAF were chosen for injection. Additionally, ‘Flanders’ strawberry plants from Yanjutian Strawberry Base, Changfeng County, Hefei City, Anhui Province, were employed, selecting those at the near-white fruit stage for injection.

### 2.7 *in situ* hybridization of pbcomt1

*In situ* hybridization was performed on 39 DAF ‘Dangshan Su’ pear fruit tissues. *In situ* hybridization involves multiple steps:

1, Tissue fixation: Pear fruit tissues were excised, rinsed, and immediately immersed in fixation solution prepared with DEPC-treated water for over 12 hours.

Dehydration and embedding: Following fixation, tissues were dehydrated through a graded ethanol series and embedded in paraffin wax.

Sectioning: Paraffin-embedded tissues were sectioned using a microtome and incubated at 62°C for 2 hours.

Dewaxing and rehydration: Sections were dewaxed in xylene I and II for 15 min each, followed by immersion in absolute ethanol I and II for 5 min each. After air-drying, sections were rehydrated in DEPC-treated water.

Enzymatic digestion: Sections were treated with 20 μg/mL proteinase K at 37°C for 22 min, rinsed with distilled water, and washed three times with PBS for 5 min each.

Pre-hybridization: A pre-hybridization solution was added and incubated at 37°C for 1 hour.

Hybridization: The pre-hybridization solution was removed, and a COMT hybridization buffer containing a 1 μM probe was added. Hybridization was carried out overnight at 42°C.

Post-hybridization washing: Slides were sequentially washed with 2× SSC at 37°C for 10 min, 1× SSC at 37°C for 5 min (twice), and 0.5× SSC at room temperature for 10 min. If nonspecific signals were observed, formamide was added to enhance specificity.

Blocking: Sections were incubated with normal rabbit serum at room temperature for 30 min.

Antibody incubation: Mouse anti-digoxigenin-conjugated alkaline phosphatase (anti-DIG-AP) was applied and incubated at 37°C for 50 min, followed by four washes in TBS for 5 min each.

Color development and mounting: BCIP/NBT substrate solution was added dropwise, and color development was monitored under a microscope. Finally, the sections were mounted with glycerol gelatin for microscopic observation.

### 2.8 Gene cloning and plant expression vector construction

To clone the gene and construct plant expression vectors, sequence-specific primers targeting *PbrCOMT1* were designed using Primer Premier 6.0 software. RT-PCR was performed with cDNA from ‘Dangshan Su’ fruit to isolate the *PbrCOMT1* gene. A *COMT1-RNAi* fragment was created using a specific *PbrCOMT1* fragment as the template.

Primers incorporating homology arms were also designed using Primer Premier 6.0. The pCAMBIA1301 vector was then digested with SmaI and SalI restriction enzymes. After digestion, the vector was ligated with the target gene using the Hieff Clone^®^ Plus One Step Cloning Kit, resulting in the construction of pCAMBIA1301-*PbrCOMT1* and pCAMBIA1301-*PbrCOMT1*-RNAi recombinant plasmids.

### 2.9 Fruit transient transformation experiments in pears and strawberries

Prepare Agrobacterium suspensions containing pCAMBIA1301-*PbrCOMT1*, pCAMBIA1301-*PbrCOMT1*-RNAi, and pCAMBIA1301-empty vectors for use with ‘Dangshan Su’ and Frankland strawberries. Collect the injected materials one week post-injection. Select a subset of fresh pear fruits and strawberries for staining observations to assess the lignin content in stone cells. Store the remaining material at -80°C for future use.

### 2.10 Genetic transformation of *PbCOMT1* overexpressing *Arabidopsis thaliana*

(1) Agrobacterium-mediated transformation of *Arabidopsis* by flower immersion:

*Agrobacterium* harboring the recombinant plasmid pCAMBIA1301- was enriched and cultured in LB liquid double antibiotic medium (Rif+ and K+) until OD_600_ reached approximately 1.0. The Agrobacterium suspension was used to infest *Arabidopsis* thaliana using the floral dip method, followed by incubation in dark conditions for 16h~24h before returning to normal light conditions and repeating the infestation weekly. This cycle was repeated three times, and seeds were collected after maturation.

(2) Screening of *PbrCOMT1* over-expressing *Arabidopsis* positive plants

Seeds were sterilized and evenly sown onto plates containing MS solid medium with chaotropic acid. Seedlings were then transferred to nutrient soil and grown in the greenhouse for about 2 weeks until four true leaves or long root whiskers had developed. Transgenic Arabidopsis plants were immersed in GUS staining solution (SL7160, Coolaber, China) at 25–37 °C for 12 hours. After staining, plants were decolorized with 70% ethanol 2–3 times until negative control tissues appeared colorless.

### 2.11 Histochemical section staining observation

Pear and strawberry fruits: Fresh fruits were cut longitudinally and stained using phloroglucinol (1% phloroglucinol staining for 5 min, followed by 18% HCl immersion for 5 min) and photographed for observation.

*Arabidopsis thaliana*: T3 generation *Arabidopsis thaliana* inflorescence axes, grown for 50 days, were selected for section observation and stained with toluidine blue (dewaxed to water and washed three times with distilled water, then soaked in 0.1% toluidine blue solution for 10 min, washed with water to remove excess staining solution, dehydrated by alcohol grading, made transparent with xylene, and sealed with neutral gum for observation).

### 2.12 Determination of stone cells and lignin in pear fruit

To assess the lignin content in ‘Dangshan Su’ fruits, 10 fruits were collected from each treatment group, and the fruit pulp was chopped and thoroughly mixed. Three 5g samples were taken from the mixture and stored at -20°C for 24 hours. The frozen samples were homogenized for three minutes at 20,000 rpm. After homogenization, distilled water was added, and the mixture was allowed to stand until the stone cells settled at the bottom of the beaker. The supernatant was carefully decanted, and this process was repeated multiple times until the upper liquid became clear. The remaining stone cells were dried and weighed. Stone cell content was calculated using the formula:


Stone cell content (%) = (weight of stone cells in g DW/weight of fruit flesh in g FW) × 100

To measure the lignin content in ‘Dangshan Su’ fruits post-injection, the pear’s skin and core were removed, and the remaining tissue was dried in an oven at 37°C. The dried material was ground into powder and passed through a 20-mesh sieve. The powder was first extracted with methanol, and the residue was dried.

Next, 0.2 g of the dried residue was weighed and extracted in 15 mL of 70% H_2_SO_4_ for 1 hour at 30°C. After the extraction, 115 mL of distilled water was added, and the solution was boiled for 1 hour, ensuring the volume remained constant. The boiled mixture was filtered using filter paper and rinsed with distilled water at 70°C until the rinse water was clear and neutral.

The remaining lignin residue was dried and weighed. All samples were analyzed in triplicate.

### 2.13 Determination of strawberry lignin

Ten strawberries from each treatment group were collected, oven-dried to a constant weight, and subsequently ground into powder. The powdered samples from each treatment group were mixed together. For lignin content analysis, five 1.0 g samples of strawberry fruit powder from each treatment group were taken. The powder was measured and mixed with 3 mL of 95% ethanol (v/v). The mixture was then centrifuged at 4°C for 10 minutes. The resulting precipitate was washed three times with 95% ethanol and three times with a 1:2 ethanol solution. To stop the reaction, 1 mL of 2 M NaOH was added, followed by 2 mL of CH_3_COOH and 1 mL of 7.5 M hydroxylamine hydrochloride. Subsequently, the mixture underwent centrifugation for 15 minutes.

Afterwards, 0.5 mL of supernatant was extracted and its absorbance was measured at 280 nm using glacial acetic acid. Lignin content was quantified using the formula: Lignin% = (Abs × volume × 100%)/(sample dry weight × standard absorbance), where:

- Lignin% denotes the percentage of lignin,
- Abs refers to the absorbance at 280 nm,
- Volume indicates the solution’s volume in liters,
- Sample dry weight is the total dry weight of the sample in grams,
- Standard absorbance is measured against the *Arabidopsis* lignin standard of 17.2.

Results were expressed as OD280 per gram. This analysis was conducted in triplicate for each sample.

### 2.14 Extraction of total plant RNA and qRT-PCR

Pear and strawberry fruits were collected one week after injection. For each group, 3–5 fruits were selected, and the injected areas were pooled as one biological replicate. Three independent biological replicates were used for each material. RNA extraction was performed using a Plant RNA Extraction Kit (V1.5, Chengdu Bafetech Co.). This RNA was subsequently converted into cDNA using the Easy Script One-Step gDNA Removal and cDNA Synthesis Super Mix Kit (Beijing All Style Gold Biotech Co.). Primers for the quantitative reverse transcription polymerase chain reaction (qRT-PCR) were designed using Primer Premier 5 software and synthesized by Sangon Biotech (Shanghai, China) (**Supplementary File 1**: **Supplementary Table S1**).

The qRT-PCR assays were conducted in a 20 μL final volume, which included 10 μL of SYBR^®^ Premix Ex Taq™ II (2X), 6.4 μL of distilled water, 0.8 μL of each primer, and 2 μL of cDNA. The microtubulin gene (AB239680.1) served as the internal control (Imai et al., 2014). Each gene was assessed with three biological replicates, and relative expression levels were calculated using the 2-ΔΔCT method as described by Livak and Schmittgen (Livak and Schmittgen, 2001).

## 3 Results

### 3.1 Identification and phylogenetic analysis of *COMT* gene

A total of 29 *COMT* genes were identified in pears for subsequent analysis. Basic information for all *COMT* genes was compiled, revealing that most *COMT* proteins exhibited pI values below 7, except for *Pbr020369.1*, which had a pI value of 9.11. The molecular weights (MW) of the COMT proteins were relatively similar, with the exception of five proteins, which had MW values below 27 kDa. The remaining 24 *COMT* proteins displayed MWs ranging from 32.35 to 43.38 kDa (**Supplementary Table S1**). Chromosomal distribution of the *COMT* genes showed a concentration in Chr10 (6 genes), followed by Chr7 (5 genes), while three *COMT* genes were distributed across Chr1, Chr10, and Chr15.

**Figure 1:**
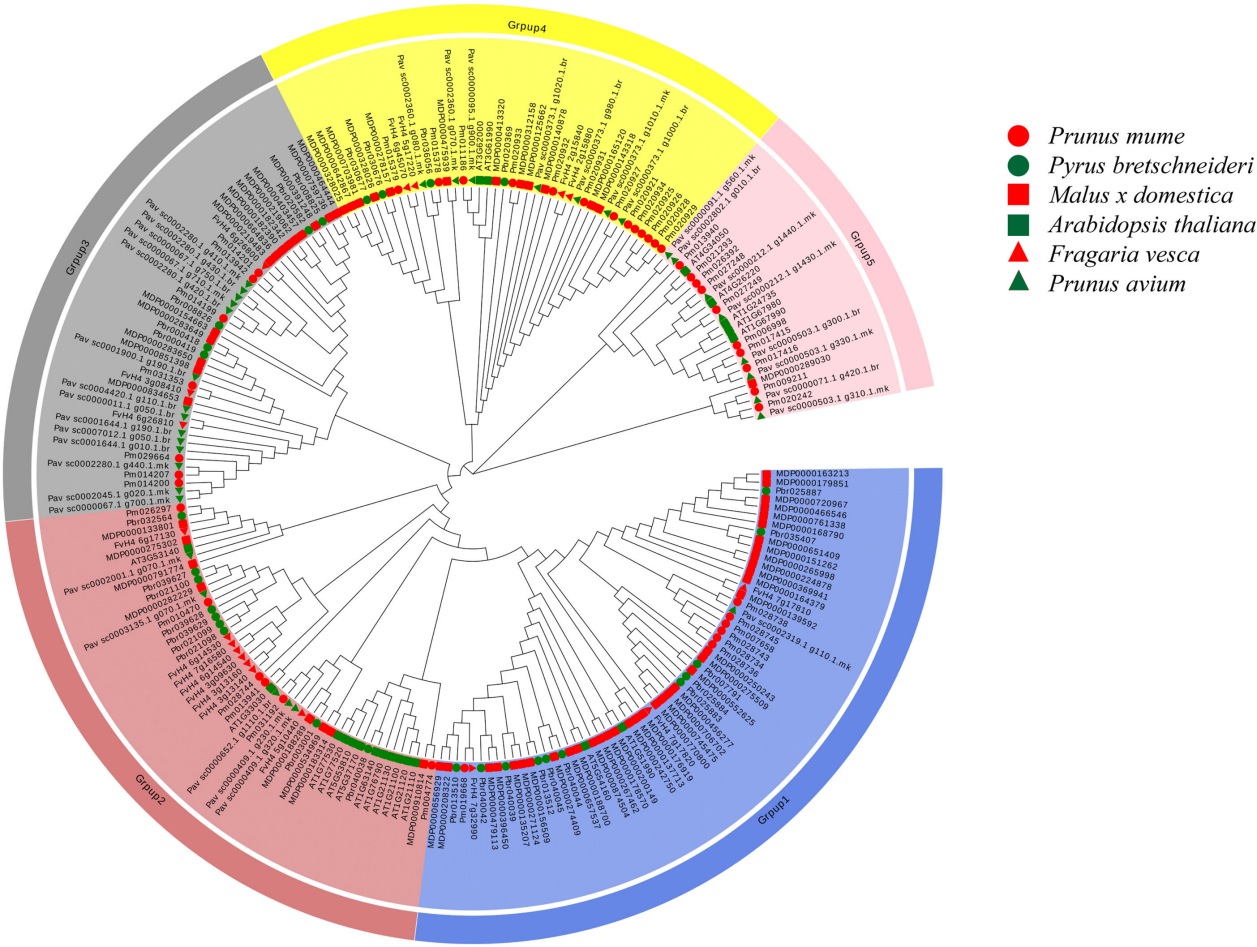
Phylogenetic tree of five Rosaceae and Arabidopsis thaliana *COMT* genes The neighbor-joining (NJ) method is used to construct the phylogenetic tree At, *Arabidopsis thaliana*; Pbr, *Pyrus bretschneideri*; Pav, *Prunus avium*; Fv, *Fragaria vesca*; MDP, *Malus x domestica*; Pm, *Prunus mume*.

A phylogenetic tree, constructed using the NJ method from 218 *COMT* proteins across *Arabidopsis* and five species of Rosaceae, including *Pyrus bretschneideri*, revealed five distinct groups (**Figure 1**). Group 1 was the largest, containing 61 *COMT* genes, including 11 from *Pyrus bretschneideri*. Group 5 included only 14 genes, with no genes from *Pyrus bretschneideri*, while Groups 2, 3, and 4 contained 48, 43, and 42 genes, respectively with 4 genes from *Pyrus bretschneideri*.

### 3.2 Structural and conserved motif analysis of *COMT* proteins

**Figure 2:**
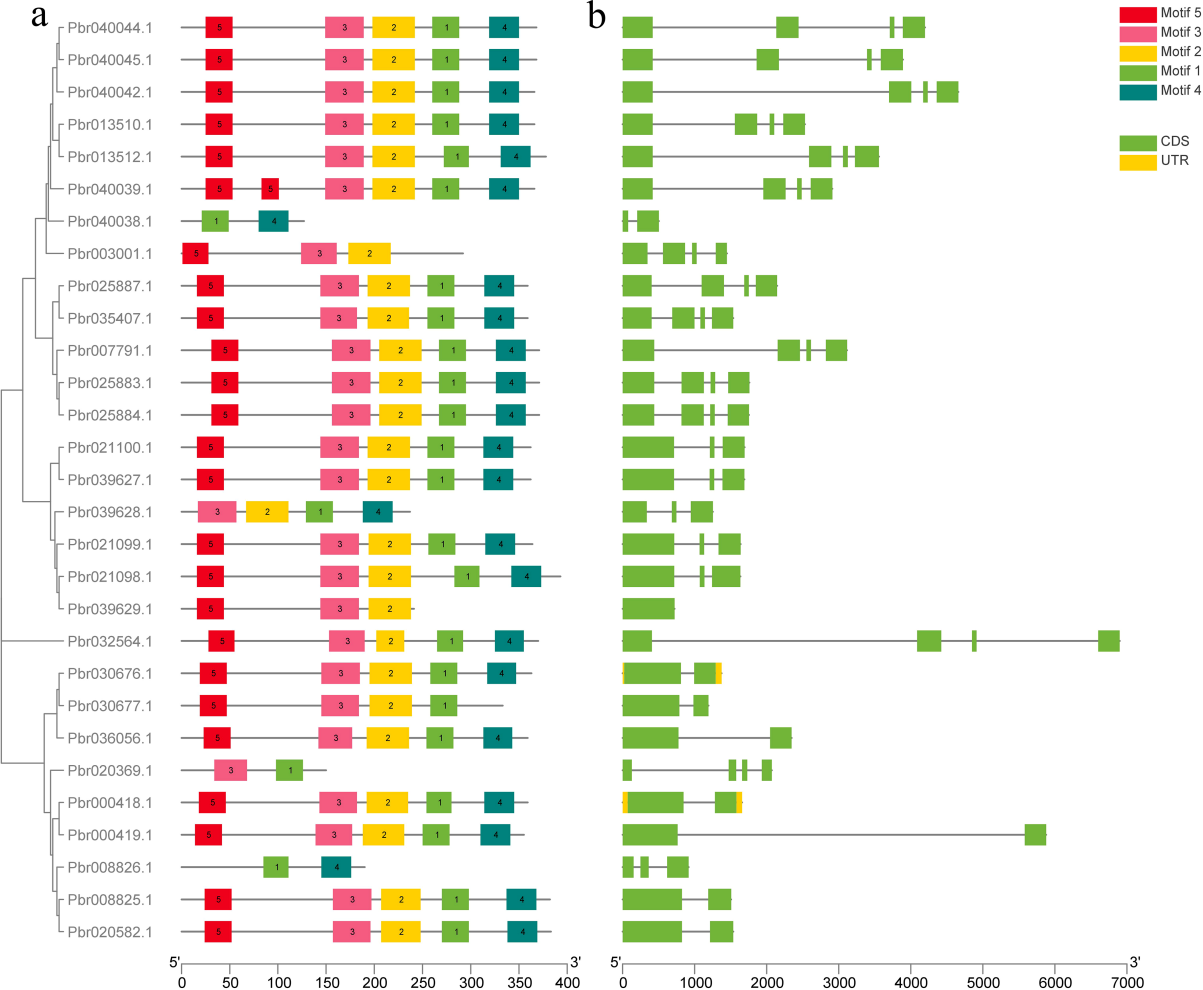
Predicted conserved motifs and gene structures of *Pyrus bretschneideri* COMT proteins. **(a)** Phylogenetic tree of *PbrCOMT* family members; **(b)** Exon-intron structure of *PbrCOMT* family members, black lines indicate introns, yellow wedges indicate exons; green wedges indicate protein coding regions.

The structural analysis of *COMT* genes in *Pyrus bretschneideri* involved generating an exon-intron map for the 29 identified *COMT* genes (**Figure 2**). All 29 genes contained motif 1, and most of them contained all five conserved motifs. Three genes (Pbr040038.1, Pbr020339.1 and Pbr008826.1) contained only two conserved motifs. Two genes (Pbr000418.1 and Pbr030676.1) had UTR regions in their sequences. The analysis of conserved structural domains is presented in **Supplementary Figure S1**.

### 3.3 Analysis of cis-acting elements in the *COMT* gene promoter

**Figure 3:**
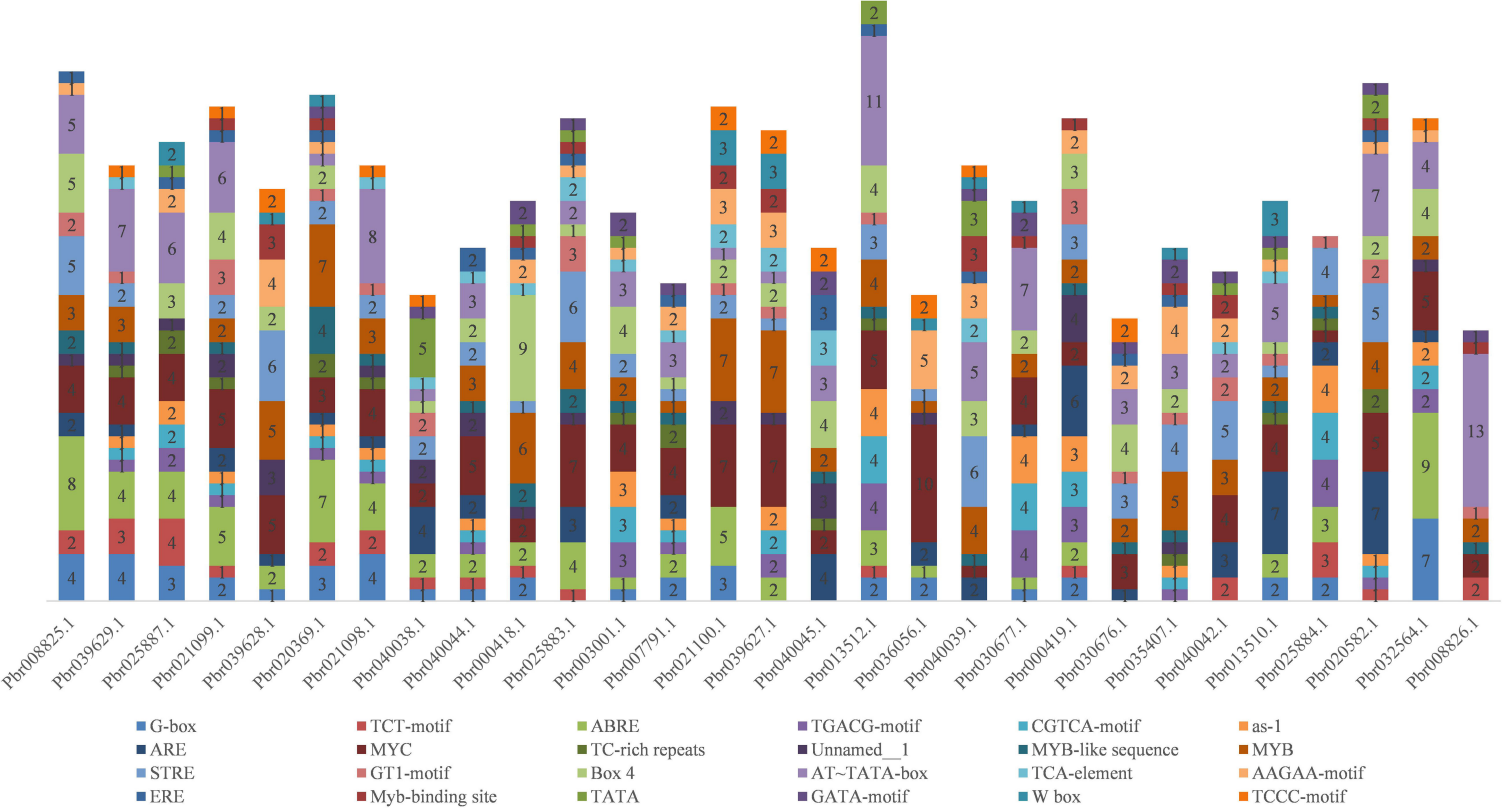
Stacking diagram of cis-acting elements in the promoter of the pear *COMT* gene. Horizontal coordinates are gene numbers, and different numbers in the graph represent the number of corresponding cis-acting elements in the promoter.

To explore the regulatory mechanisms of the *COMT* genes, cis-acting elements in the promoters of 29 *PbrCOMT* genes from *Pyrus bretschneideri* were predicted (**Figure 3**). The MYC element was the most prevalent, with 115 MYC cis-acting elements identified across the 29 *COMT* genes, except for Pbr035407.1. Light-responsive elements were found in all promoters, including G-box, Box 4, GT1-motif, GATA-motif, and TCCC-motif. Hormone-responsive cis-acting elements were also abundant, including TGACG and CGTCA motifs responsive to methyl jasmonate (MeJA), TCA-element responsive to salicylic acid, and ABRE, which responds to abscisic acid. Additionally, cis-acting elements such as TC-rich repeats and ARE were identified, which are involved in stress and defense responses, as well as anaerobic induction. The analysis also indicated several MYB binding sites in the promoters, suggesting that MYB transcription factors may play a significant role in regulating lignin synthesis in *Pyrus bretschneideri*.

### 3.4 Expression pattern analysis of the *PbCOMT1* gene

**Figure 4:**
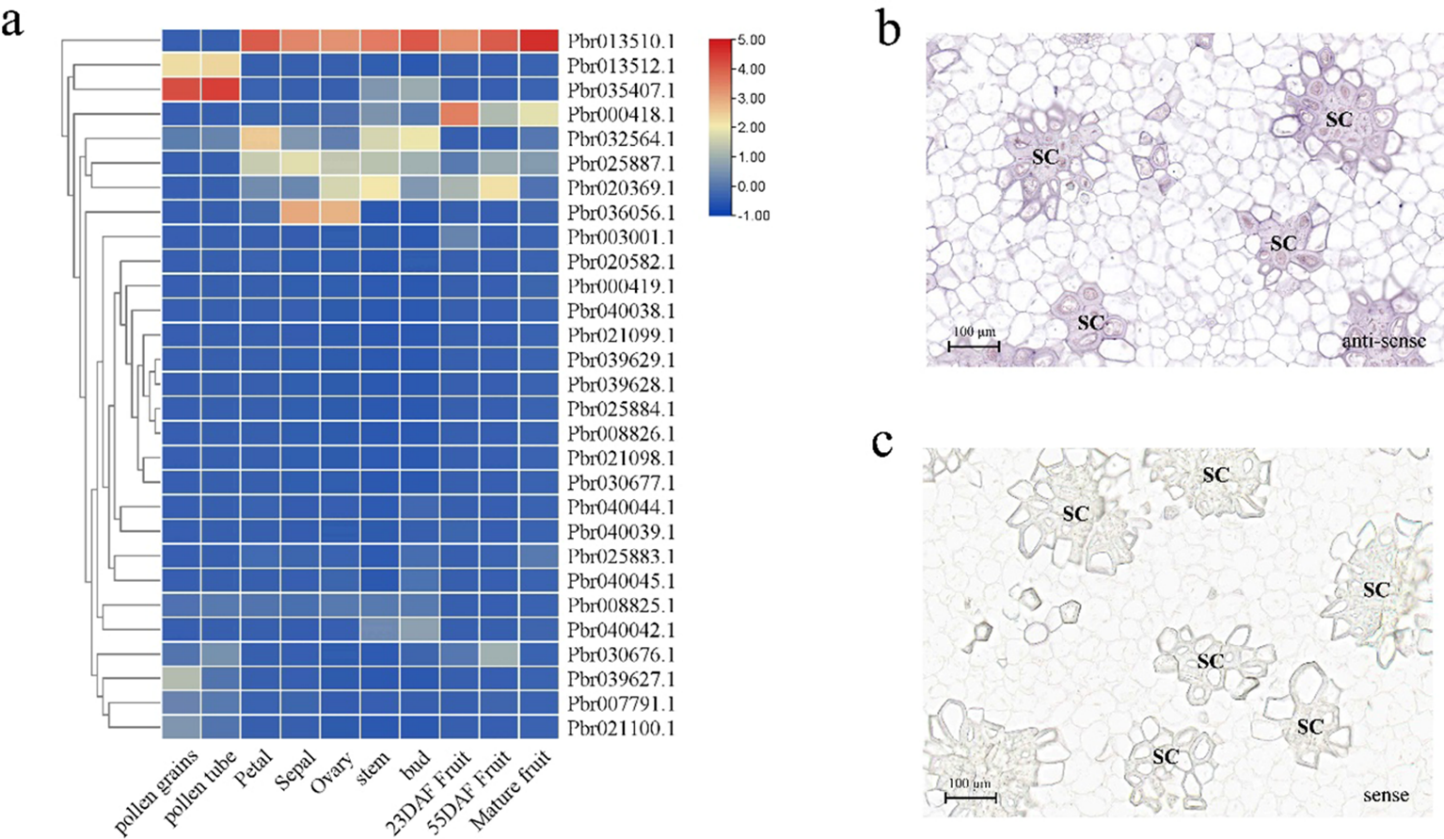
Expression profiles of *COMT* family genes in the fruit of ‘DangShan Su’. **(a)** Heatmap of *COMT* family gene expression in different tissue types and developmental stages of pear fruit. *In situ* hybridization results of *PbrCOMT1* antisense **(b)** and sense **(c)** RNA probes in pear fruit at 39 DAF. SC, stone cells.

The expression profiles of the *PbCOMT* genes in ‘DangShan Su’ were analyzed using transcriptome data from different tissue types and developmental stages of pear fruit. *Pbr013512.1* and *Pbr035407.1* showed higher expression levels in pollen grains and pollen tubes, indicating their central role in pollen maturation and development. In other tissues such as petal, sepal, ovary, stem, and bud, five genes (*Pbr013510.1, Pbr032564.1, Pbr025887.1, Pbr020369.1*, and *Pbr036056.1*) exhibited higher expression levels compared to other genes. In pear fruit at different developmental stages, *Pbr013510.1* and three other genes (*Pbr000418.1*, *Pbr025887.1*, *Pbr020369.1*) showed relatively high expression levels at various stages. Notably, Pbr013510.1 exhibited consistently high expression in all tissues and developmental stages of pear fruit, except for pollen grains and pollen tubes, and its expression was significantly higher than that of other *COMT* family genes. This suggests that Pbr013510.1 is the primary functional *COMT* gene in these tissues and developmental stages of pear fruit (**Figure 4a**, **Supplementary Table S2**).

To further investigate the role of the *COMT* gene family in *Pyrus bretschneideri*, *Pbr013510.1* (designated *PbrCOMT1*) was selected for further functional validation. RNA *in situ* hybridization was performed on fresh ‘Dangshan Su’ fruit collected 39 days post-flowering to explore the correlation between *PbrCOMT1* expression and lignin deposition and SCW thickening. The positive expression of *PbrCOMT1* transcripts was visualized using BCIP/NBT staining, which produced a blue-purple coloration. *In situ* hybridization with antisense probes confirmed that *PbrCOMT1* transcripts were localized in specific regions of the pulp cell walls and in clusters of stone cells within the pear fruit (**Figure 4b**). As a control, the sense probe showed no significant staining in the pear fruit, indicating that *PbrCOMT1* is involved in both lignin synthesis and the formation of stone cells (**Figure 4c**).

### 3.5 Histochemical staining observations of *PbrCOMT1* transiently transformed pear fruit

**Figure 5:**
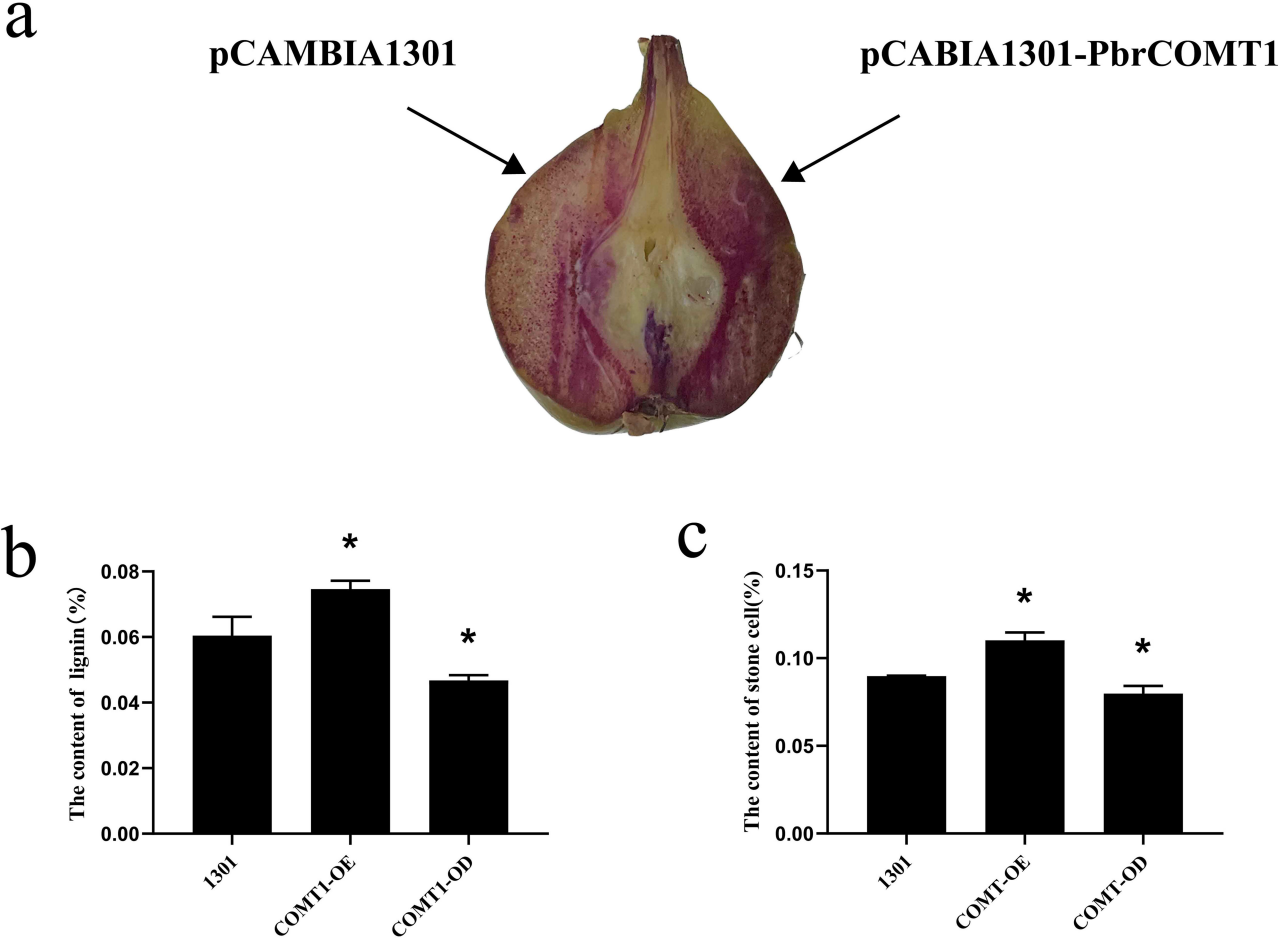
Analysis of *PbrCOMT1* in 39DAF pear fruit. **(a)** Staining plots of *PbrCOMT1* transiently transformed pear fruits after mesotrione staining. **(b)** Stone cell statistics of *PbrCOMT1* transiently transformed pear fruit. **(c)** Lignin content statistics of *PbrCOMT1* transiently transformed pear fruit. COMT-OE: Instantaneous overexpression of *PbrCOMT1* in pear fruit, COMT-OD: Instantaneous Silence *PbrCOMT1* Pear Fruit,* indicates *P*<0.05, the bar = 100 µm.

Fresh pear fruit samples, injected with either Pcambia1301- *PbrCOMT1* or pCAMBIA1301 empty vector, were subjected to phloroglucinol staining. The staining patterns revealed significant differences between the two treatments. The pear fruit injected with pCAMBIA1301-*PbrCOMT1* exhibited a noticeably darker staining on one side compared to the empty vector control, suggesting a higher lignin content (**Figure 5a**).

### 3.6 Comparative analysis of the stone cell and lignin content in pear fruit derived from COMT-OE and COMT-OD

To further investigate the effects of transient transformation on pear fruit, we quantified the lignin and stone cell content in the transformed samples. The results showed that the lignin content in COMT-OE pear fruit was 7.4 ± 0.25%, representing a 23% increase compared to the 6.0 ± 0.57% observed in the pCAMBIA1301 empty vector control. In contrast, the lignin content in COMT-OD fruit was 4.6 ± 0.16%, approximately 76% of the control level (**Figure 5b**). Similarly, stone cell content was higher in COMT-OE fruit (11 ± 0.46%), showing a 22% increase compared to the control (8.97%), while COMT-OD fruit contained 7.98% stone cells, about 89% of the control level (**Figure 5c**). These findings indicate that the transient overexpression of *PbrCOMT1* promotes lignin biosynthesis and increases stone cell content in pear fruit.

### 3.7 Expression pattern analysis of essential enzyme genes involved in lignin biosynthesis in transiently transformed pear fruit

**Figure 6:**
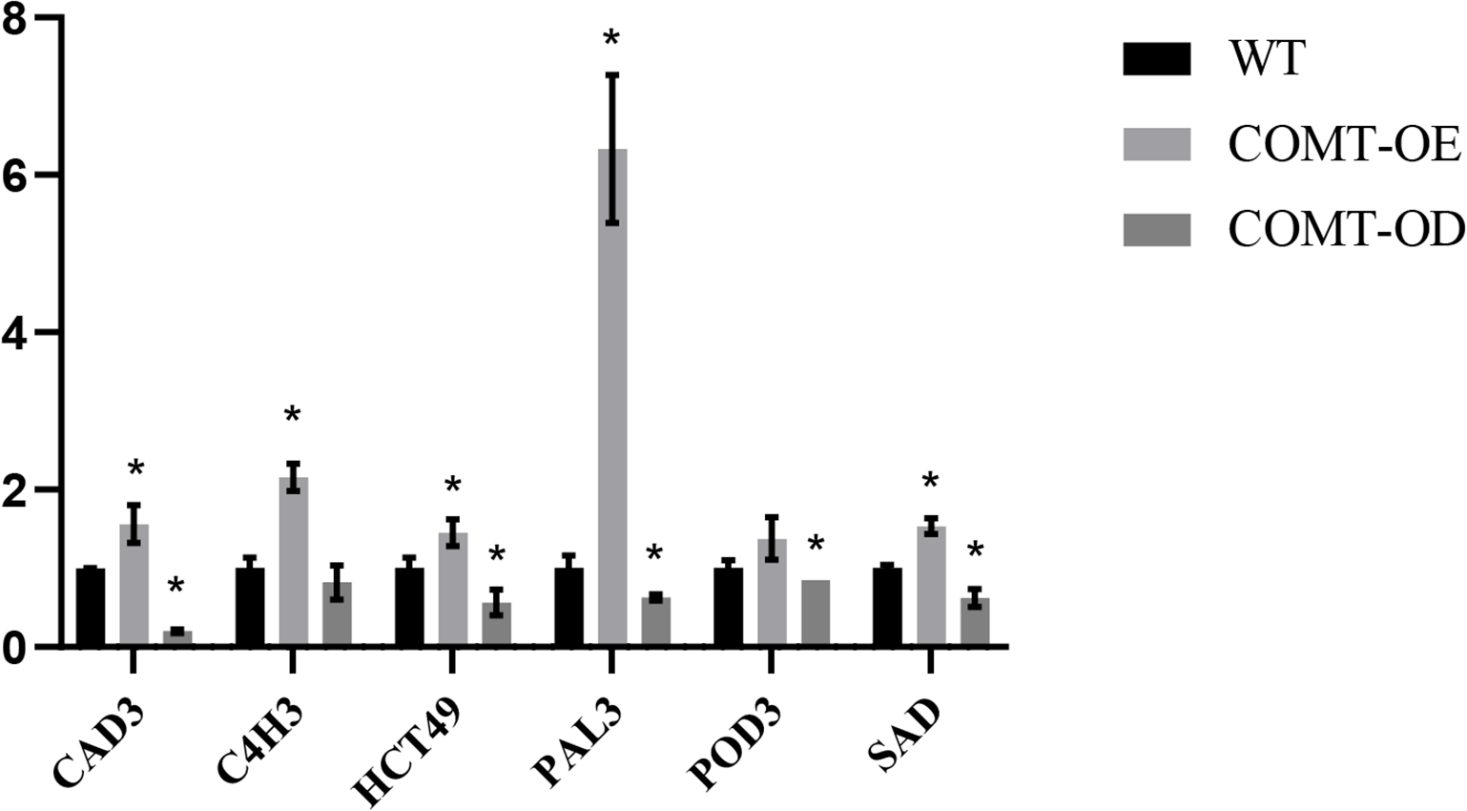
Analysis of the expression patterns of key lignin synthesis enzyme-encoding genes in *PbrCOMT1*-overexpressing pear fruits. COMT-OE indicates pear fruits overexpressing *PbrCOMT1*, COMT-OD indicates pear fruits in which *PbrCOMT1* is silenced. * denotes *P*<0.05.

We further examined the expression patterns of key structural genes involved in lignin biosynthesis in COMT-OE and COMT-OD pear fruits using fluorescence quantification. The results revealed that the overexpression of *PbrCOMT*1 significantly upregulated the expression of several essential lignin biosynthesis genes, including *CAD3*, *C4H3*, *HCT49*, *PAL3*, *POD3*, and *SAD*. Notably, *PbrPAL3* exhibited the most substantial increase, with its expression level elevated by 6.33-fold. In contrast, the expression of lignin biosynthesis genes in COMT-OD pear fruit was considerably reduced, with *CAD3* expression only reaching 0.19-fold of that in the wild type (WT) (**Figure 6**).

### 3.8 Staining and lignin content analysis of strawberry plants transiently transformed with *PbrCOMT1*

**Figure 7:**
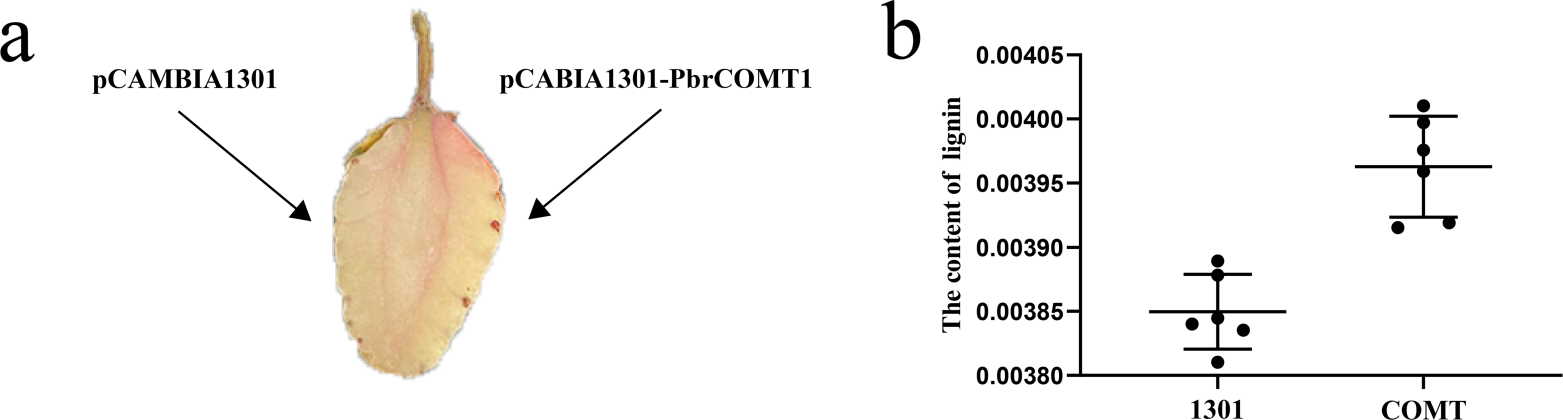
Transient transformation of strawberry fruit by *PbrCOMT1*. **(a)** Staining of strawberry fruits with resorcinol **(b)** Statistics of lignin content in strawberry fruits.

To further validate the function of *PbrCOMT1*, we transiently transformed strawberry plants from the Rosaceae family, specifically the ‘Yuexiu’ variety at the white fruit stage. Phloroglucinol staining revealed that the overexpression of *PbrCOMT1* resulted in a much darker red staining compared to the control, indicating a significant increase in lignin content in the strawberries (**Figure 7a**). Lignin content was quantitatively assessed, showing that strawberries injected with the pCAMBIA1301 empty vector contained approximately 0.385% lignin. In contrast, strawberries overexpressing *PbrCOMT1* exhibited a slight increase in lignin content to approximately 0.396% (**Figure 7b**).

### 3.9 Plant height statistics of *PbrCOMT1*-transformed *Arabidopsis* with mutants

**Figure 8:**
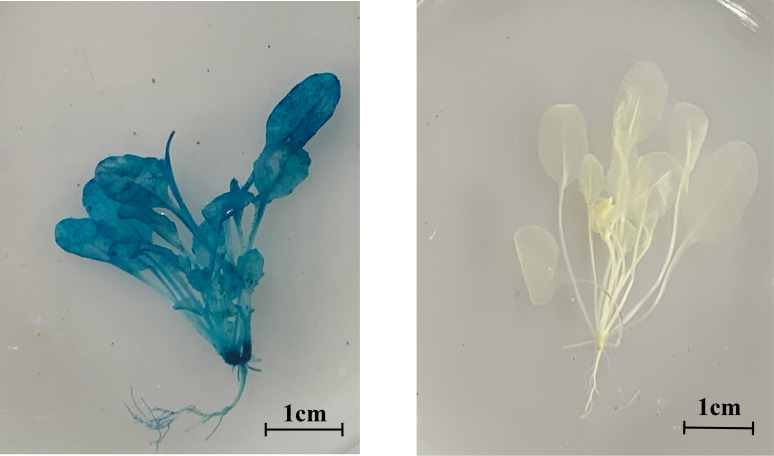
Arabidopsis GUS staining, left, overexpression of *PbrCOMT1*, right, WT.

**Figure 9:**
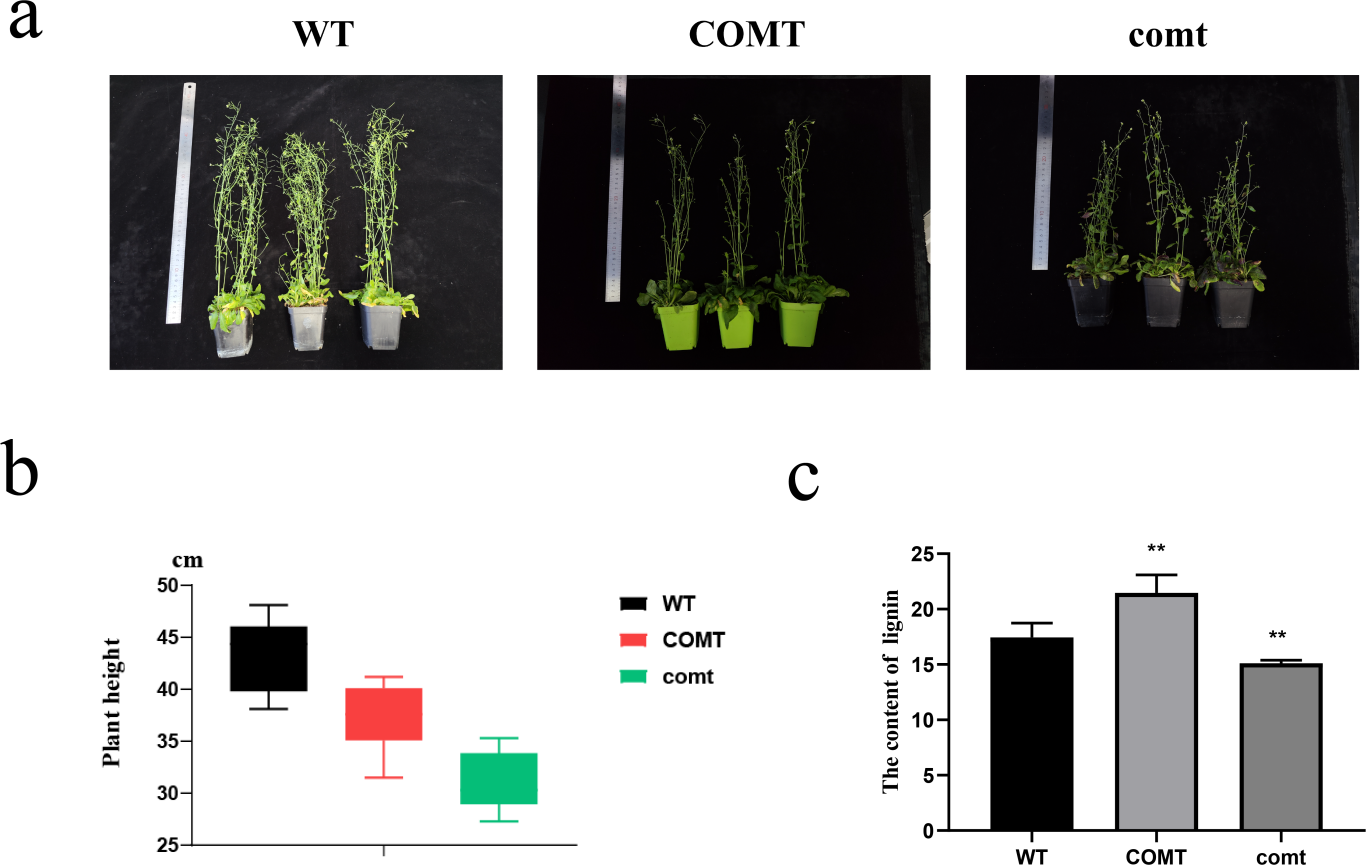
Growth status and lignin statistics of Arabidopsis overexpressing *PbrCOMT1* and *PbrCOMT1* mutant Arabidopsis thaliana **(a)** Pictures of 50-d-old *Arabidopsis thaliana* plants. **(b)** Average plant height statistics of *Arabidopsis thaliana* plants. **(c)** Lignin content statistics of Arabidopsis thaliana. wt: wild-type *Arabidopsis thaliana*, COMT: *PbrCOMT1* overexpressing T3 generation *Arabidopsis thaliana*; comt: *PbrCOMT1* homozygous *Arabidopsis thaliana* mutant line. ** indicates *P*<0.01.

To confirm the successful transformation of *PbrCOMT1*-GUS into *Arabidopsis* thaliana, we performed GUS staining on T3 generation plants. The COMT (*PbrCOMT1*-overexpressing) *Arabidopsis* plants were visibly stained blue, while the WT plants remained almost transparent, confirming successful transformation (**Figure 8**). Plant height measurements taken after 50 days of growth showed that the average height of WT plants was 43.4 cm, whereas the height of COMT and comt mutant plants was reduced to 37.3 and 31.0 cm respectively, in comparison to the WT (**Figure 9b**).

### 3.10 Changes in lignin content of *PbrCOMT1*-transformed *Arabidopsis* and mutants

**Figure 10:**
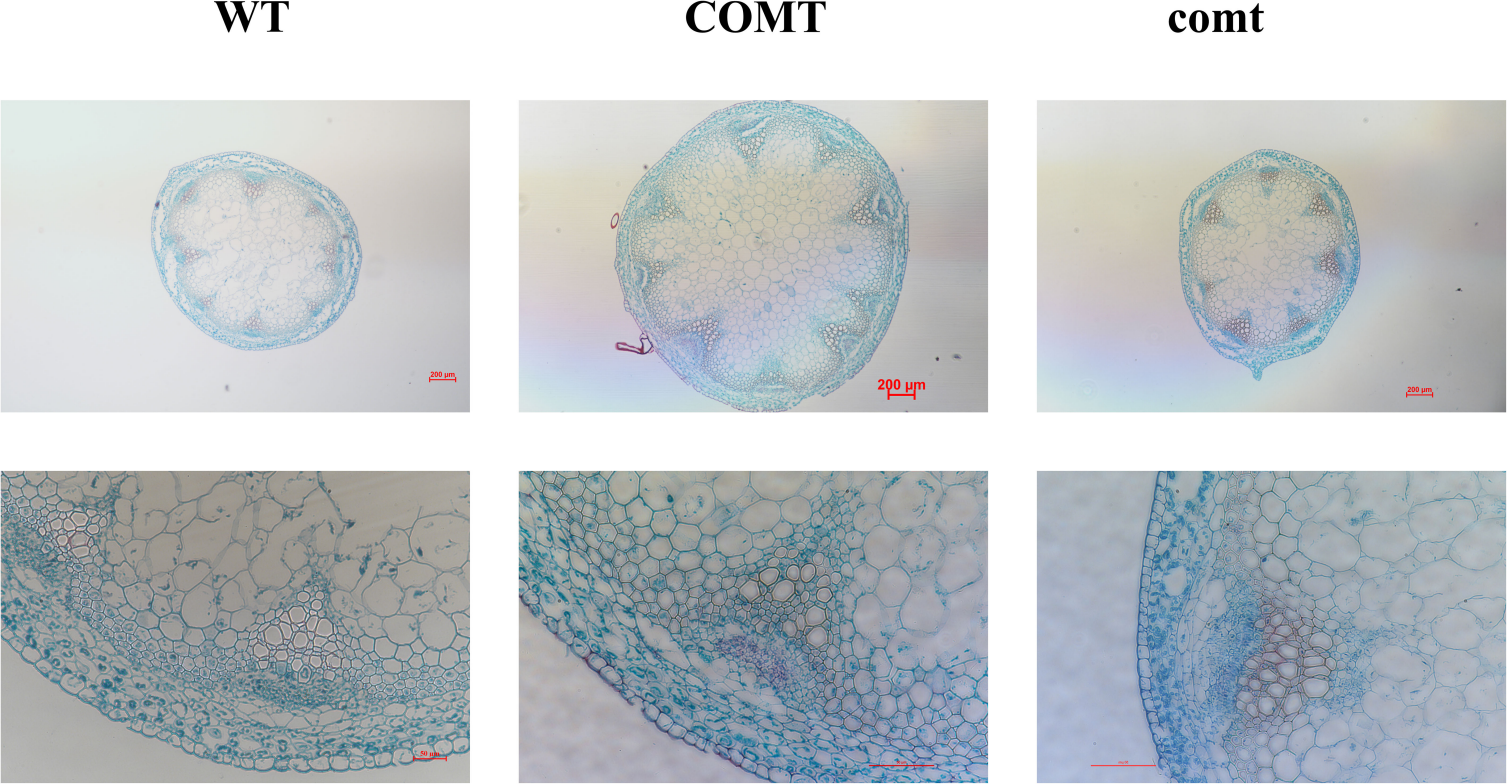
Toluidine blue staining plots of inflorescence axes of *PbrCOMT1* overexpressing Arabidopsis and mutants. wt: wild-type Arabidopsis, COMT: *PbrCOMT1* overexpressing T3-generation Arabidopsis; comt: *PbrCOMT1* homozygous Arabidopsis mutant lines.

*Arabidopsis* has been stably transformed with *PbrCOMT1* to investigate its role in lignin synthesis. Toluidine blue staining on wild-type (WT), *COMT*-overexpressing (COMT), and *COMT*-deficient (comt) *Arabidopsis* plants visualized lignin distribution within the inflorescence axis. Staining results showed that *COMT*-overexpressing plants displayed more intense staining in the xylem and interbundle fibers, with a notable increase in stem diameter compared to WT plants. In contrast, *COMT*-deficient mutants exhibited less intense staining. Quantitative analysis of lignin content using the acetyl bromide method revealed a 23% increase in *COMT*-overexpressing plants and a 13% decrease in comt mutants compared to WT (**Figure 10**).

## 4 Discussion

Genes and protein structures, and most *PbrCOMT* genes share the same motif, indicating similar protease functions. However, analyses of *PbrCOMT* promoters revealed significant differences; some are regulated by hormones, while others respond to external stress. A large number of these promoters are influenced by transcription factors, suggesting that functionally identical genes may respond differently to stimuli, facilitating varied functions in plants. Drought, MeJA, and ABA have been shown to enhance *CmCOMT* expression in melon (Liu et al., 2021), whereas MeJA also promotes *COMT* expression in Hibiscus cannabinus (Kim et al., 2013), tobacco (Toquin et al., 2003), and *Arabidopsis* (Joshi et al., 2022). Furthermore, *COMT* is regulated by transcription factors, and MYB has been identified as a regulator of *COMT* expression in maize (Velez-Bermudez et al., 2015), willowherb (Alexander et al., 2020), *Arabidopsis* (Kim et al., 2020), and blueberry (Yang et al., 2022).

Upon examining the three-dimensional structure of the *PbrCOMT1* protein, it was found to be a dimer, which may underlie its methylation function. The dimerization site of *COMT* proteins likely occurs at the C-terminus, and each monomer contains a ligand that catalyzes SAH/SAM (Singh and Sharma, 2022).

A significant increase in lignin content was observed in *PbrCOMT1*-transformed pear fruits. However, the increase in *PbrCOMT1*-transiently transformed strawberry fruits was not substantial, possibly due to the inherently low lignin content of strawberries and the low expression of other lignin synthesis genes (Yeh et al., 2014). In these strawberry fruits, the expression of other lignin genes limited the synthesis rate, and the low expression of upstream and downstream genes in the lignin synthesis pathway resulted in insufficient substrates and the accumulation of products, thereby reducing the overall synthesis rate.

When overexpressing and silencing *PbrCOMT*1 in pear fruit, consistent trends were observed in the content of lignin and stone cells. This consistency is attributed to the concentration of lignin in the stone cells of pears, where an increase in lignin content accelerates SCW production and promotes stone cell generation (Gong et al., 2020; Xu et al., 2021).

In *Arabidopsis*, inconsistent phenotypes of inflorescence axes were observed following overexpression and silencing of *COMT*, although both modifications followed similar trends in plant height. Overexpression of *COMT* may increase the lignin content of the plant stalk, inhibiting growth, while silencing *COMT* could result in insufficient lignin for proper conduit development, thus impeding plant growth and affecting the development of *Arabidopsis*. It has been reported that either excessive or insufficient lignin can restrict plant growth (Ha et al., 2021; Xie et al., 2018), likely due to a balance between defense mechanisms and growth, which when disrupted by external factors, leads to the plant prioritizing defense over growth.

## 5 Conclusion

A total of 29 *PbrCOMTs* were screened in pears, revealing structural similarities but diverse cis-acting elements in their promoters. *In situ* hybridization studies demonstrated that *PbrCOMT1* is associated with stone cell development in pear fruits. Following the transient transformation of *PbrCOMT1*, an increase in both stone cell and lignin content was observed in pear fruits. Conversely, silencing *PbrCOMT1* resulted in decreased stone cell and lignin contents, while overexpression of *PbrCOMT1* enhanced lignin production in both strawberry and *Arabidopsis thaliana*. *PbrCOMT1* has been identified as a key gene in lignin synthesis in ‘Dangshan Su’ pears, suggesting its role in promoting lignin synthesis and stone cell production, thereby providing a theoretical foundation for enhancing the quality of pear fruits.

## Data Availability

The genomic data used in this study, including CDSs, protein sequences, and gene annotation files (in GFF/GFF3 format), are publicly available. The transcriptome data for this experiment are deposited in the SRA database under the accession numbers SRR5965142, SRR5965144, and SRR5965146, accessible at https://www.ncbi.nlm.nih.gov/sra. Transcriptome annotation was performed using the pear genome data, which can be accessed at https://www.ncbi.nlm.nih.gov/datasets/genome/GCF_000315295.1/.
