# Supplementary material for: Functional characterization of COMT genes in Chinese white pear (Pyrus bretschneideri) and their role in lignin synthesis
Source: Front Plant Sci. 2025 Jun 9;16:1614220. doi: 10.3389/fpls.2025.1614220 (PMC12183264; doi:10.3389/fpls.2025.1614220)
Supplement: Supplementary file 1 [file DataSheet1.docx]

Table S1: 29 *COMT* genesBasic information in *Pyrus bretschneideri*

| Gene ID | Chromosome | Start (bp) | End (bp) | AA | MW(kDa) | pI | Pfam |
| --- | --- | --- | --- | --- | --- | --- | --- |
| *Pbr000418.1* | Chr5 | 25491960 | 25493619 | 358 | 39.69 | 5.50 | Pfam:Methyltransf_2 |
| *Pbr000419.1* | Chr5 | 25483797 | 25489678 | 354 | 39.65 | 5.51 | Pfam:Methyltransf_2 |
| *Pbr003001.1* | scaffold1122.0 | 23106 | 24554 | 291 | 32.35 | 6.16 | Pfam:Methyltransf_2 |
| *Pbr007791.1* | Chr1 | 1297791 | 1300908 | 370 | 41.03 | 5.78 | Pfam:Methyltransf_2 |
| *Pbr008825.1* | scaffold1546.0 | 20658 | 22166 | 381 | 42.58 | 5.90 | Pfam:Methyltransf_2 |
| *Pbr008826.1* | scaffold1546.0 | 26275 | 27192 | 189 | 21.59 | 5.18 | Pfam:Methyltransf_2 |
| *Pbr013510.1* | Chr1 | 10266961 | 10269493 | 365 | 39.74 | 5.63 | Pfam:Methyltransf_2 |
| *Pbr013512.1* | Chr1 | 10260971 | 10264530 | 377 | 41.00 | 5.67 | Pfam:Methyltransf_2 |
| *Pbr020369.1* | Chr6 | 3179841 | 3181913 | 149 | 16.48 | 9.11 | Pfam:Methyltransf_2 |
| *Pbr020582.1* | Chr8 | 9326537 | 9328073 | 382 | 42.52 | 5.31 | Pfam:Methyltransf_2 |
| *Pbr021098.1* | Chr15 | 599973 | 601608 | 392 | 43.38 | 6.14 | Pfam:Methyltransf_2 |
| *Pbr021099.1* | Chr15 | 603813 | 605452 | 363 | 40.19 | 6.08 | Pfam:Methyltransf_2 |
| *Pbr021100.1* | Chr15 | 607471 | 609165 | 361 | 40.02 | 5.59 | Pfam:Methyltransf_2 |
| *Pbr025883.1* | Chr10 | 11072707 | 11074468 | 370 | 40.97 | 5.96 | Pfam:Methyltransf_2 |
| *Pbr025884.1* | Chr10 | 11092007 | 11093762 | 370 | 41.00 | 6.10 | Pfam:Methyltransf_2 |
| *Pbr025887.1* | Chr10 | 11141041 | 11143186 | 358 | 39.00 | 5.70 | Pfam:Methyltransf_2 |
| *Pbr030676.1* | Chr9 | 19942101 | 19943476 | 362 | 39.94 | 6.10 | Pfam:Methyltransf_2 |
| *Pbr030677.1* | Chr9 | 19945337 | 19946529 | 332 | 36.50 | 5.78 | Pfam:Methyltransf_2 |
| *Pbr032564.1* | Chr12 | 10376444 | 10383345 | 369 | 40.53 | 5.26 | Pfam:Methyltransf_2 |
| *Pbr035407.1* | scaffold684.0 | 92066 | 93602 | 358 | 39.48 | 5.28 | Pfam:Methyltransf_2 |
| *Pbr036056.1* | Chr17 | 17443507 | 17445853 | 358 | 39.39 | 5.38 | Pfam:Methyltransf_2 |
| *Pbr039627.1* | Chr10 | 6133378 | 6135069 | 361 | 40.02 | 5.40 | Pfam:Methyltransf_2 |
| *Pbr039628.1* | Chr10 | 6127577 | 6128831 | 236 | 26.51 | 6.25 | Pfam:Methyltransf_2 |
| *Pbr039629.1* | Chr10 | 6124652 | 6125374 | 240 | 26.50 | 6.08 | Pfam:Methyltransf_2 |
| *Pbr040038.1* | Chr7 | 14706144 | 14706648 | 126 | 13.76 | 6.27 | Pfam:Methyltransf_2 |
| *Pbr040039.1* | Chr7 | 14698217 | 14701130 | 365 | 39.59 | 5.75 | Pfam:Methyltransf_2 |
| *Pbr040042.1* | Chr7 | 14680634 | 14685296 | 365 | 39.63 | 5.88 | Pfam:Methyltransf_2 |
| *Pbr040044.1* | Chr7 | 14631481 | 14635679 | 367 | 40.05 | 5.63 | Pfam:Methyltransf_2 |
| *Pbr040045.1* | Chr7 | 14601663 | 14605555 | 367 | 39.87 | 5.82 | Pfam:Methyltransf_2 |

Table S2: FPKM values for COMT family genes in different tissues and developmental stages of pear fruit

| GeneID | pollen grains | pollen tube | Petal | Sepal | Ovary | stem | bud | 23DAF Fruit | 55DAF Fruit | Mature fruit |
| --- | --- | --- | --- | --- | --- | --- | --- | --- | --- | --- |
| *Pbr008825.1* | 0.15 | 0.17 | 0.39 | 0.61 | 1.02 | 0.62 | 0.63 | 0.00 | 0.00 | 0.00 |
| *Pbr039629.1* | 0.00 | 0.00 | 0.00 | 0.00 | 0.00 | 0.00 | 0.00 | 0.00 | 0.00 | 0.00 |
| *Pbr025887.1* | 0.00 | 0.00 | 5.61 | 35.62 | 11.64 | 3.89 | 2.62 | 0.40 | 7.73 | 1.72 |
| *Pbr021099.1* | 0.00 | 0.00 | 0.00 | 0.01 | 0.00 | 0.00 | 0.00 | 0.00 | 0.00 | 0.00 |
| *Pbr039628.1* | 0.00 | 0.00 | 0.00 | 0.00 | 0.00 | 0.00 | 0.00 | 0.00 | 0.00 | 0.00 |
| *Pbr020369.1* | 0.00 | 0.00 | 1.10 | 2.10 | 16.06 | 8.87 | 1.54 | 2.10 | 74.02 | 0.30 |
| *Pbr021098.1* | 0.00 | 0.00 | 0.01 | 0.00 | 0.00 | 0.01 | 0.02 | 0.00 | 0.00 | 0.00 |
| *Pbr040038.1* | 0.00 | 0.00 | 0.00 | 0.05 | 0.00 | 0.00 | 0.00 | 0.00 | 0.00 | 0.00 |
| *Pbr040044.1* | 0.00 | 0.00 | 0.03 | 0.02 | 0.09 | 0.01 | 0.26 | 0.00 | 0.00 | 0.00 |
| *Pbr000418.1* | 0.00 | 0.00 | 0.07 | 0.22 | 0.67 | 1.46 | 0.63 | 19.85 | 13.08 | 9.41 |
| *Pbr025883.1* | 0.00 | 0.00 | 0.16 | 0.26 | 0.21 | 0.01 | 0.37 | 0.00 | 0.14 | 0.54 |
| *Pbr003001.1* | 0.00 | 0.00 | 0.00 | 0.01 | 0.01 | 0.03 | 0.00 | 0.63 | 0.00 | 0.00 |
| *Pbr007791.1* | 0.31 | 0.17 | 0.04 | 0.23 | 0.07 | 0.11 | 0.15 | 0.00 | 0.26 | 0.00 |
| *Pbr021100.1* | 0.48 | 0.13 | 0.01 | 0.10 | 0.00 | 0.00 | 0.00 | 0.00 | 0.00 | 0.00 |
| *Pbr039627.1* | 0.97 | 0.13 | 0.01 | 0.06 | 0.00 | 0.00 | 0.00 | 0.00 | 0.00 | 0.00 |
| *Pbr040045.1* | 0.00 | 0.00 | 0.00 | 0.07 | 0.28 | 0.00 | 0.45 | 0.00 | 0.00 | 0.00 |
| *Pbr013512.1* | 1.99 | 1.68 | 0.01 | 0.09 | 0.15 | 0.07 | 0.00 | 0.00 | 0.03 | 0.00 |
| *Pbr036056.1* | 0.00 | 0.00 | 0.19 | 240.02 | 81.47 | 0.00 | 0.00 | 0.00 | 0.00 | 0.07 |
| *Pbr040039.1* | 0.00 | 0.00 | 0.02 | 0.03 | 0.01 | 0.03 | 0.16 | 0.05 | 0.04 | 0.00 |
| *Pbr030677.1* | 0.00 | 0.00 | 0.00 | 0.00 | 0.01 | 0.00 | 0.02 | 0.00 | 0.00 | 0.00 |
| *Pbr000419.1* | 0.00 | 0.00 | 0.00 | 0.00 | 0.03 | 0.02 | 0.03 | 0.00 | 0.00 | 0.05 |
| *Pbr030676.1* | 0.16 | 0.35 | 0.00 | 0.03 | 0.02 | 0.01 | 0.25 | 0.34 | 8.99 | 0.00 |
| *Pbr035407.1* | 5.63 | 4.74 | 0.06 | 0.09 | 0.15 | 1.49 | 2.36 | 0.00 | 0.00 | 0.00 |
| *Pbr040042.1* | 0.00 | 0.00 | 0.00 | 0.01 | 0.10 | 1.10 | 1.86 | 0.00 | 0.07 | 0.06 |
| *Pbr013510.1* | 0.00 | 0.00 | 74.81 | 463.60 | 142.23 | 33.68 | 43.90 | 17.03 | 1418.67 | 198.63 |
| *Pbr025884.1* | 0.00 | 0.00 | 0.00 | 0.00 | 0.00 | 0.00 | 0.00 | 0.00 | 0.00 | 0.00 |
| *Pbr020582.1* | 0.00 | 0.00 | 0.05 | 0.00 | 0.03 | 0.00 | 0.07 | 0.00 | 0.00 | 0.00 |
| *Pbr032564.1* | 0.24 | 0.26 | 16.75 | 3.87 | 1.28 | 5.70 | 7.04 | 0.00 | 0.02 | 0.43 |
| *Pbr008826.1* | 0.00 | 0.00 | 0.00 | 0.00 | 0.00 | 0.00 | 0.00 | 0.00 | 0.00 | 0.00 |

Motif 1
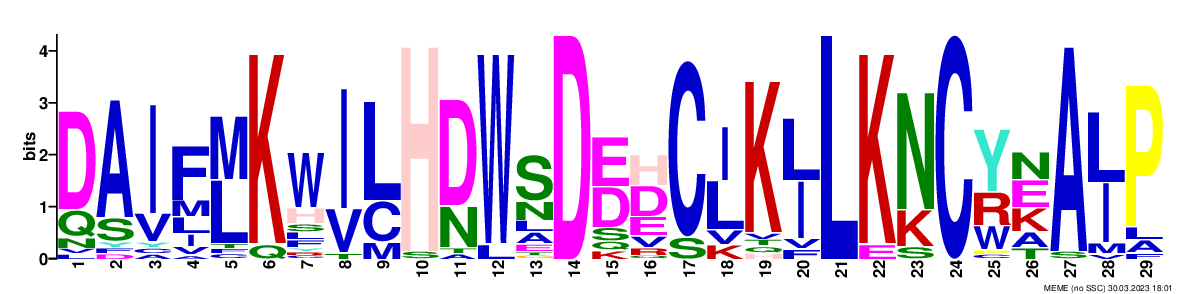


Motif 2


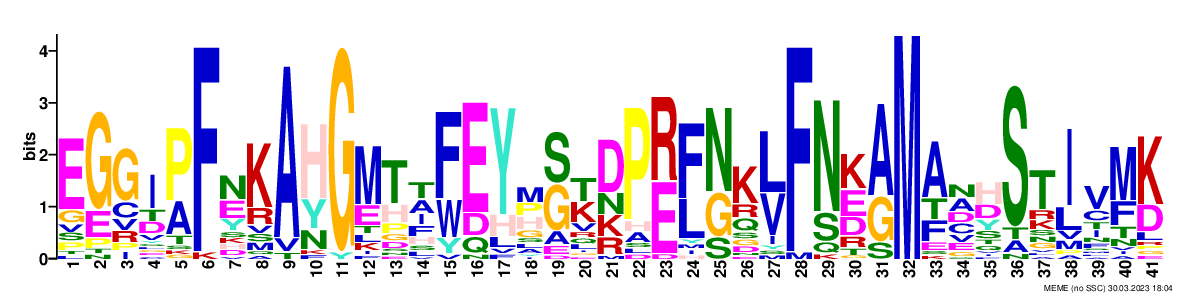


Motif 3


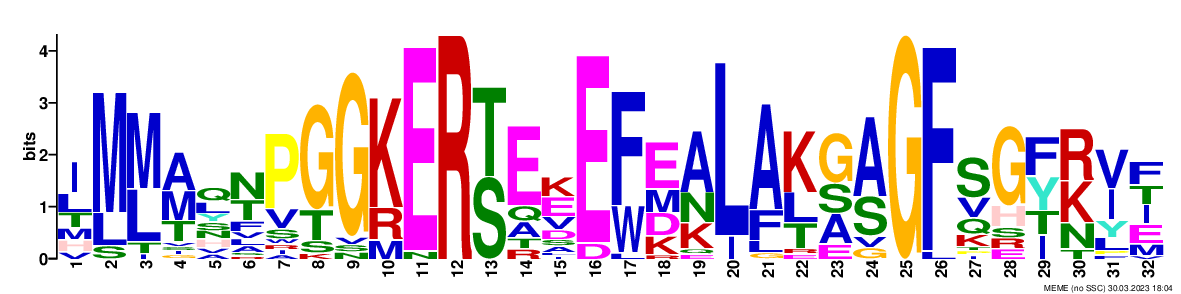
Motif 4
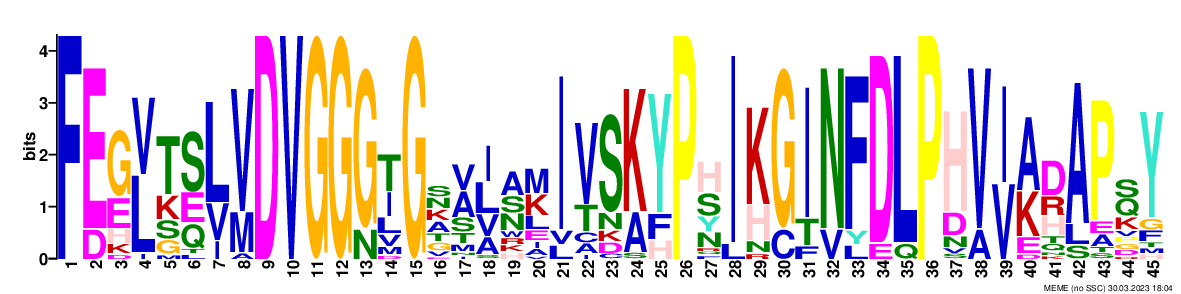


Motif 5


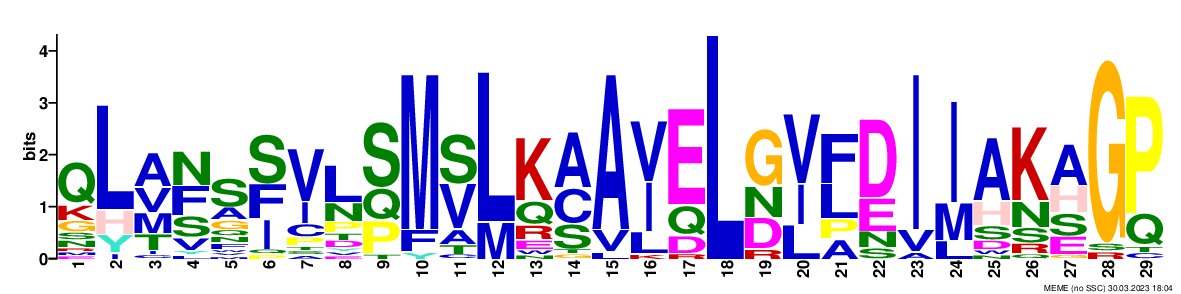


Fig.S1 5 motifs of the COMT genes


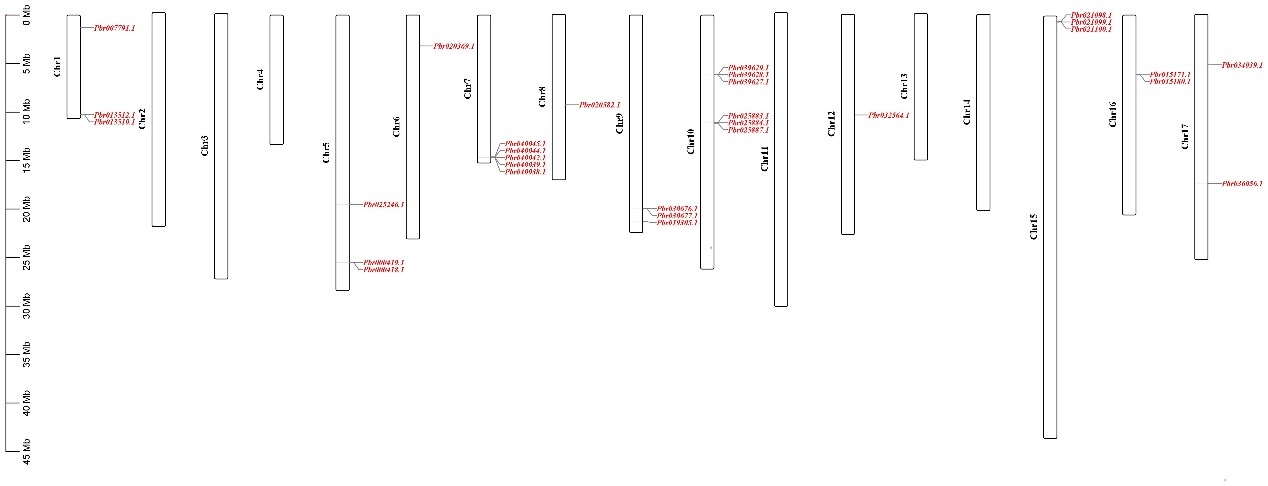


Fig.S2 *Pyrus bretschneideri* Pbrcomt chromosome localization
